# Light-driven reversible charge transfers from ITO nanocrystals

**DOI:** 10.3389/fchem.2023.1288681

**Published:** 2023-11-02

**Authors:** Luca Rebecchi, Andrea Rubino, Andrea Camellini, Ilka Kriegel

**Affiliations:** ^1^ Functional Nanosystems, Istituto Italiano di Tecnologia, Genova, Italy; ^2^ Dipartimento di Chimica e Chimica Industriale, Università degli Studi di Genova, Genova, Italy; ^3^ Department of Mechanical Engineering, Columbia University, New York, NY, United States

**Keywords:** indium tin oxide, nanocrystals, photodoping, hole acceptors, charge transfer

## Abstract

The combination of semiconductors and redox active molecules for light-driven energy storage systems has emerged as a powerful solution for the exploitation of solar batteries. On account of this, transparent conductive oxide (TCO) nanocrystals (NCs) demonstrated to be interesting materials, thanks to the photo-induced charge accumulation enabling light harvesting and storage. The charge transfer process after light absorption, at the base of the proper use of these semiconductors, is a key step, often resulting in non-reversible transformations of the chemicals involved. However, if considering the photocharging through TCO NCs not only as a charge provider for the system but potentially as part of the storage role, the reversible transformation of the redox compound represents a crucial aspect. In this paper, we explore the possible interaction of indium tin oxide (ITO) NCs and typical redox mediators commonly employed in catalytic applications with a twofold scope of enhancing or supporting the light-induced charge accumulation on the metal oxide NC side and controlling the reversibility of the whole process. The work presented focuses on the effect of the redox properties on the doped metal oxide response, both from the stability point of view and the photodoping performance, by monitoring the changes in the optical behavior of ITO/redox hybrid systems upon ultraviolet illumination.

## Introduction

**FIGURE 1:**
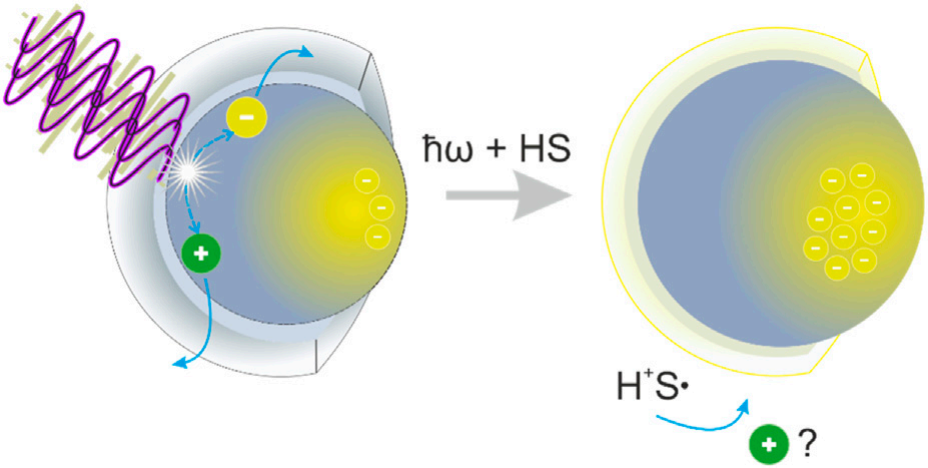
Illustration of the photodoping process in one single ITO NC. After light absorption with light beyond the bandgap (*ħω* ≥ E_g_), an electron–hole pair is created. If the hole reacts with a hole scavenger (HS), the electron remains within NC to increase the carrier density.

The concept of reversibility in energy transformation is crucial for sustainable development. Technological progress aims at systems capable of effectively using resources without compromising their availability, and for this purpose, the concepts of recycling and reuse both in terms of process and materials become fundamental (Winkler, 2011). With regards to the conversion of renewable sources, sunlight is one of the most promising prospects. Still, its use is based mostly on its abundance and worldwide availability and, therefore, on a rather ineffective consumption. A system capable of performing the function of direct and reversible electrochemical storage of solar energy would be an ideal solution (Ghini et al., 2021a; Lv et al., 2018; Moseley and Garche, 2014; Kharkats and Pleskov, 1994). However, if the materials used for absorption and storage are different and disjointed, the whole process will inevitably suffer irreversible losses (Podjaski and Lotsch, 2021). One of the most interesting recent solutions concerning the integration of solar conversion with electrochemical storage is that of redox flow solar batteries (Wedege et al., 2018). Such systems function as a rechargeable photoelectrochemical cell, in which the photo-generated charges from a semiconductor can be transferred to a redox couple in a solution. In a configuration of this type, the relationship between the light absorption (optoelectronics) and the faradic charge storage (electrochemistry) becomes crucial (Schoetz et al., 2022). Many organic redox couples are currently in use for flow batteries, depending on parameters such as stability, solubility, and reversibility (Cao et al., 2018). On the function instead of active materials for photoelectrodes, wide bandgap semiconductors are the undisputed protagonists. However, a more specific class of nanometric compounds made of doped metal oxides stands out among the others for an additional degree of manipulation of the light–matter interaction. Indeed, some of these oxides have demonstrated the possibility of absorbing photons above the bandgap, generating charges and separating them cut out increasing their charge density (Kriegel et al., 2020). This phenomenon is also known as photodoping because the accumulation of charge carriers occurs primarily, thanks to illumination (Figure 1). However, the latter is not the only necessary condition. In order to support such a phenomenon, the material must be able to delocalize the charges and neutralize those of opposite sign (Das et al., 2022). One of the most studied examples is that of tin-doped indium oxide (ITO) nanocrystals (NCs), able of accumulating hundreds of electrons through exposure to ultraviolet light (Reynal et al., 2013; Rebecchi et al., 2023). However, to counterbalance this effect, a material that can accept the photo-generated holes, a hole scavenger (HS), is normally needed (Ghini et al., 2021b). The separation of the electron–hole pairs limits the loss of charges and, therefore, of energy by recombination. To optimize this characteristic quantitatively and qualitatively, it is necessary to further investigate the function of holes quenching to increase the charge density and maintain this charge under non-illumination conditions. On one hand, the extraction of holes using an irreversible scavenger can significantly improve the effect of photodoping and make the use of excess electrons more effective for further reactions. Such conditions are a typical prerogative of photocatalysis, one of the most popular applications for the use of the photoresponse of similar compounds (Reynal et al., 2013; Denisov et al., 2019). On the other hand, considering the possibility to re-use the metal oxide, in order to bring the material back to a pre-exposure state, an irreversible extraction of the excess electrons is, in principle, sufficient, for example, by bringing the NCs back into contact with atmospheric oxygen (Joost et al., 2018). Nevertheless, with a view to a more effective technological use, the presence of sacrificial electron-donor or electron-acceptor compounds becomes undesirable as they would need to be continuously replaced with fresh material (Reynal et al., 2013). To use these photoactive metal oxides in combination with electrochemical storage, instead, it would be ideal to adopt the active redox couples of the flow batteries as hole collectors, albeit carefully considering the working conditions, such as to avoid, for example, unwanted reactions that could lead to the recombination of the charges. Moreover, the best use of redox mediators, in this aspect, should imply multitasking. These compounds can serve as hole acceptors, supporting the accumulation of the photo-generated electrons on the semiconductors, but they can also act on charge storage as in a flow battery configuration or even as a countermeasure to keep under control the recovery of the initial conditions allowing recycling of the system.

In this work, we analyzed the possibility of merging and taking advantage of two different properties, hole scavenging and redox reversibility, by combining the ITO NCs with three of the most used species in flow cell batteries, (2,2,6,6-tetramethylpiperidin-1-yl)oxyl (TEMPO), crystal violet, and ferrocene (Fc) (Armstrong et al., 2020), as reversible hole scavengers, and observed their effect on the photodoping of the ITO NCs. Particular focus was laid on understanding the reversibility and the effect of enhancing the charge storing properties of ITO NCs under illumination.

For this reason, in this work, we have focused the analysis on the case of ITO NCs and their photo-induced electron accumulation property as they represent a highly attractive bifunctional material for solar energy storage (Kriegel et al., 2020; Ghini et al., 2021a).

## Materials and methods

The synthesis of indium tin oxide (ITO) NCs was carried out, according to the following procedure. Indium (III) acetate (CAS: 25,114-58-3), tin (IV) acetate (CAS: 2800-96-6), oleic acid (technical grade, 90% purity, CAS: 112-80-1), and oleyl alcohol (technical grade, 85% purity, CAS: 143-28-2) were procured from Sigma-Aldrich. In the initial step, 13 mL of oleyl alcohol was loaded in a 100-mL three-neck round-bottom flask and degassed for 3 h at 150°C, under a nitrogen atmosphere. Meanwhile, 263 mg of indium and 45 mg of tin precursors, along with 2 mL of oleic acid, were combined in a 50-mL three-neck round-bottom flask. Under continuous stirring, the flask content was degassed for 3 h under a nitrogen flux, leading to the formation of tin and indium oleates. After degassing, the flask containing oleyl alcohol, serving as the reaction vessel, was maintained under a nitrogen flux of 0.130 L/min and heated to 290°C. The indium and tin precursors were transferred into a syringe and injected into the hot oleyl alcohol at a controlled rate of 0.3 mL/min using a syringe pump. NC growth continued for 15 min after the injection concluded. Synthesis was then cooled to room temperature. The resulting solution was then subjected to centrifugation at 5540 G for 10 min, with ethanol used as an antisolvent. The supernatant was discarded, and the material was redispersed in hexane. Subsequently, ethanol was added again, and the solution underwent a second round of centrifugation under the same parameters. Finally, the synthesized NCs were stored in octane. An ITO NC stock solution was prepared by taking an aliquot of NCs, which was dried up and brought into a glovebox, to be redispersed in anhydrous hexane with a final concentration of 9.5 mg/mL. Hole scavenger stock solutions were prepared using anhydrous hexane (Sigma-Aldrich) as the solvent, with a concentration of 7.4 mg/mL for TEMPO (CAS: 2564-83-2, Sigma-Aldrich) solutions, 20 mg/mL for ferrocene (CAS: 102-54-5, Sigma-Aldrich), and 1 mg/mL for crystal violet (CAS: 548-62-9, Sigma-Aldrich) solutions. All dispersions were prepared in an argon-filled glovebox to avoid any contact with atmospheric oxygen.

The photodoping of ITO NCs was carried out by dispersing them in anhydrous hexane (Sigma-Aldrich) and sealing the dispersion in an optical cuvette made of Infrasil, with an optical path of 2 mm (Starna Scientific). To obtain the absorption spectra, 17 µL of ITO NCs were dispersed in 700 µL of anhydrous hexane and transferred in an optical cuvette. All quantities were tuned to obtain well-defined spectra without signal saturation. UV light was then used to illuminate the cuvette from UV LED (Thorlabs M300L4, central wavelength: 300 nm, bandwidth: 20 nm) placed in a box internally coated with aluminum foil, placed 12 mm away from the external cuvette window. The effects of TEMPO, crystal violet, and ferrocene on ITO NC photodoping dynamics were studied by adding, respectively, 50 µL (TEMPO), 10 µL (crystal violet), or 10 µL (ferrocene) stock solution to 700 µL of anhydrous hexane, along with 17 µL of ITO NC stock solution in hexane. A normal photodoping procedure was then followed, as discussed previously.

Absorption spectra (Agilent Cary 5,000) to monitor the optical response of the ITO NC solutions were measured upon the addition of the hole scavengers, both before and after exposure to UV light. The concentration and chemical composition of NCs were estimated by inductively coupled plasma mass spectrometry (ICP-OES), while dimensions and morphology were characterized by transmission electron microscopy (TEM, JEOL JEM-1400Plus - Analytical 120 kV TEM/STEM).

## Results and discussion

### ITO NC photodoping

**FIGURE 2:**
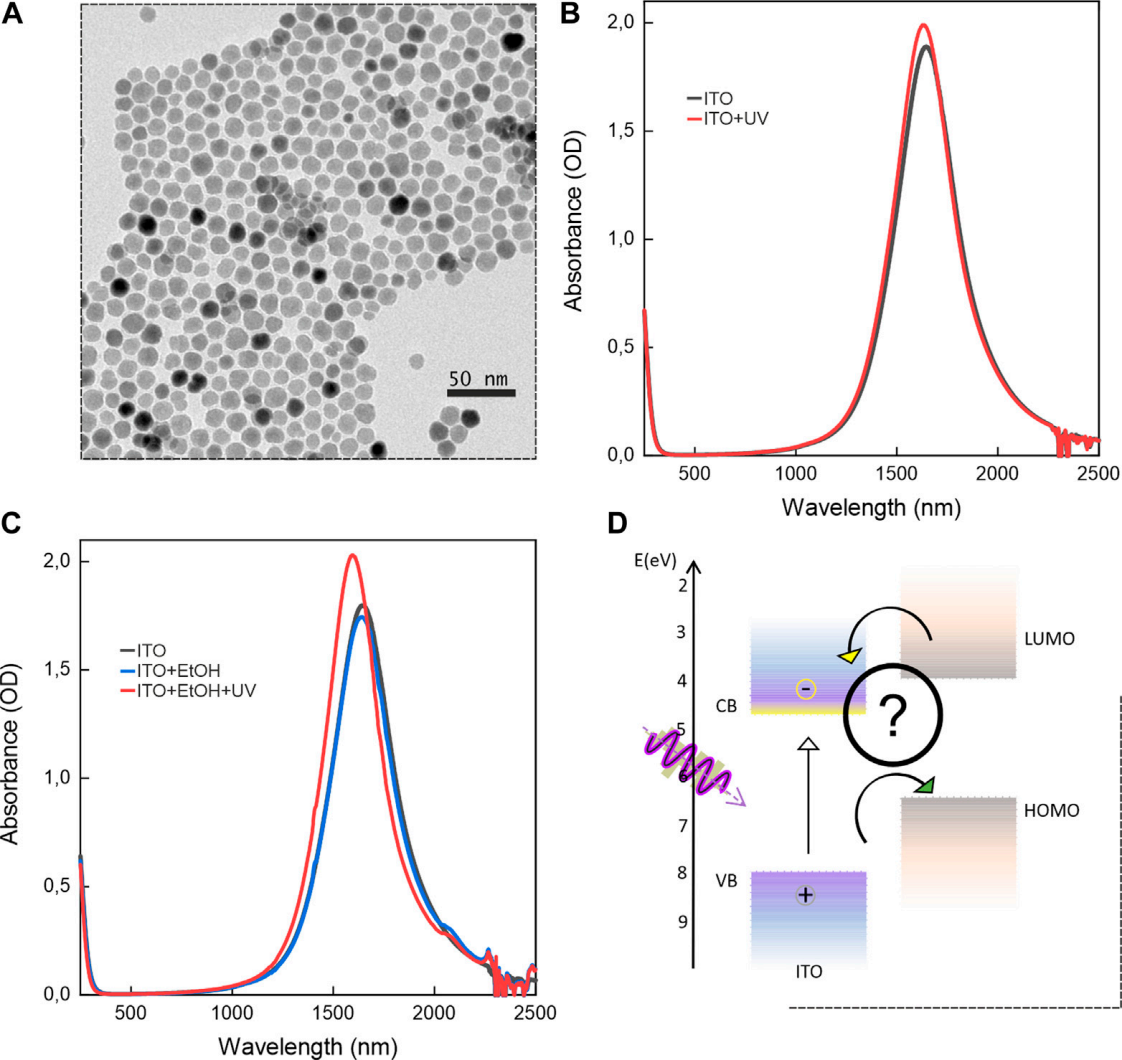
**(A)** Typical TEM image of ITO NCs. **(B)** Black curve: ITO NCs before UV light illumination. Red curve: ITO NCs after UV light illumination. The black curve represents the typical absorption spectrum of ITO NC dispersion in hexane. In the NIR, at approximately 1700 nm, the LSPR peak is present, which is the result of tin aliovalent doping, while the bandgap is found in the UV range. Upon photodoping, the plasmonic peak increases in intensity and shifts the position toward shorter wavelengths. **(C)** Photodoping in the presence of a common hole acceptor, such as ethanol, displays an enhanced and more efficient photodoping, as observed by the more intense changes in the plasmon peak. This effect is illustrated with the evolution from the blue to red curve. The reaction with ethanol, however, results in an irreversible transformation. **(D)** Possible energy-level alignment of a suitable redox reversible hole scavenger.

The ITO NCs used in this study have a pseudospherical shape, with an average diameter of approximately 13 nm (Figure 2A). Figure 2B shows the typical absorption spectrum of ITO NCs, displaying the main features of the material, which are the bandgap in the UV spectral region, the localized surface plasmon resonance (LSPR) in the near-infrared window (NIR) region, approximately 1,700 nm, and the visible transparency. To first analyze the progress of photodoping, we monitored the evolution of the LSPR peak of the ITO NC solution in hexane. The LSPR spectral position and intensity are, in fact, dependent on the density of free charge carriers, which increases upon light-induced charge accumulation (Rebecchi et al., 2023). In particular, photodoping will induce a blue shift and intensity increase (Rebecchi et al., 2023). Figure 2B shows how ITO NCs in solution, alone, can undergo photodoping. Indeed, comparing the absorption spectrum of ITO before and after UV light exposure, it can be seen both a blue shift in position and intensity increase of the LSPR peak. Typically, sacrificial hole scavengers (HS) play an essential role in the photodoping process of colloidal NCs, contributing to the efficient generation of charge carriers. Photodoping of different materials has been found in the literature, such as titanium nanoparticles (Joost et al., 2018) and zinc iron oxide (ZIO) (Brozek et al., 2018). These have been tested with a variety of hole scavengers such as methanol (Wei et al., 2019), ethanol (Joost et al., 2018; Armstrong et al., 2020; Ghini et al., 2021b), and triethanolamine hydrochloride (TEA) (Zayats et al., 2003). In all cases reported, stable charge accumulation can only be achieved, upon switching off the ultraviolet illumination, and by adding HS. Nonetheless, the role of a hole quencher can also be played by other elements in the system. The solvent, for example, can help in hole scavenging as for the case of alcohols (Eglitis et al., 2020). Different ligands also demonstrated the possibility to act as a hole acceptor for the negative photocharging of semiconductor NCs (Hu et al., 2019a; Hu et al., 2019b; Mic et al., 2020). However, photo-generated holes can follow different paths if no hole scavenging occurs. One is hole trapping, which can occur on intrinsic defects or trap states within the NC structure (i.e., oxygen vacancies) (Gierster et al., 2022). Under such conditions, the photo-generated charges can incur non-radiative recombination mechanisms like trap-assisted or Auger recombination (Cohn et al., 2012) and phonon-induced energy release. These competing alternative routes will hinder the photo-generated electron accumulation process.

In the literature, there are reports about the photodoping of ZnO and FICO (fluorine indium cadmium oxide). Indeed, ZnO can photodope only in the presence of a hole quencher, as a consequence of its very fast recombination dynamics (Cohn et al., 2012). Similarly, FICO can only accumulate electrons if photo-generated holes are consumed (Kriegel et al., 2016) with a hole-quenching compound (e.g., ethanol). In our case, we observe photodoping in the absence of HS, as shown in Figure 2B. We speculate that it might be a consequence of the concomitant effects of oleate ligand passivation (usually characterized by fast trapping/de-trapping equilibrium) (Yan et al., 2021), defects in the lattice (oxygen vacancies) (Gan et al., 2013), and surface states in the depletion region (Ghini et al., 2022).

As a reference for our proposed mechanism, we decided to test the photodoping effect with the support of a well-known hole scavenger, ethanol (Figure 2C). The presence of this molecule, even a small amount, increases the photodoping efficiency of ITO NCs. In the first place, in Figure 2C, it is shown how the ITO spectrum from the as-prepared state state (black curve) is not substantially modified upon adding 30 µL of ethanol (EtOH) to the cuvette (blue curve). Under this condition, we can safely analyze the ITO behavior. Then, comparing the ITO/EtOH mixture before (blue curve) and after UV exposure (red curve), it is shown how ITO photodoping is enhanced, as compared to the case with no EtOH. Indeed, with the addition of EtOH, the LSPR peak intensity has increased by 16%, while without EtOH, photodoped plasmon resonance has increased by 5.3%. For the sake of clarity, we show this difference in Supplementary Figure S1, where we compare the photodoping process using the normalized LSPR absorbance of ITO NCs and ITO NCs with the addition of ethanol, both in the as-prepared and photodoped states for similar exposure time (15 min). In any case, this increase in efficiency comes at the cost of irreversibly oxidizing EtOH molecules. In the literature, it is reported that upon exposure to photodoped NCs (Katsiev et al., 2017), ethanol oxidizes to acetaldehyde, following the two-step mechanism described in Eqs 1–3. The process typically involves two of the photo-generated holes in two successive reactions. The first one induces the formation of a hydroxyethyl radical, which can subsequently trap the second hole for the complete oxidation (Katsiev et al., 2017). In addition, as counter evidence of the ethanol transformation upon photodoping, we were able to observe the changes in the spectral region surrounding the absorption peak typical of the stretching of the -OH group of the ethanol molecule. It is demonstrated in Supplementary Figures S2A, B how this peak’s intensity decreases after photodoping, proving that ethanol was consumed during the process in favor of the formation of its oxidized species (NIST, 2023).

$$ITO+\hbar \omega \;\to \;e^{-}\left(CB\right)+h^{+}\left(VB\right),$$ (1)

$${CH}_{3}{CH}_{2}OH+h^{+}\to \;{CH}_{3}\dot{C}HOH+H^{+},$$ (2)

$${CH}_{3}\dot{C}HOH+h^{+}\to \;{CH}_{3}CH=O+H^{+}.$$ (3)

As already mentioned, the aim of this study is to exploit the same mechanism to enhance photodoping while retaining the ability to recover pre-illumination conditions, thus regenerating the hole-scavenging molecules and ITO NCs. The ideal candidates are molecules that can undergo reversible redox reactions while having a favorable energy-level alignment with the band structure of ITO NCs (Figure 2D).

### TEMPO

**FIGURE 3:**
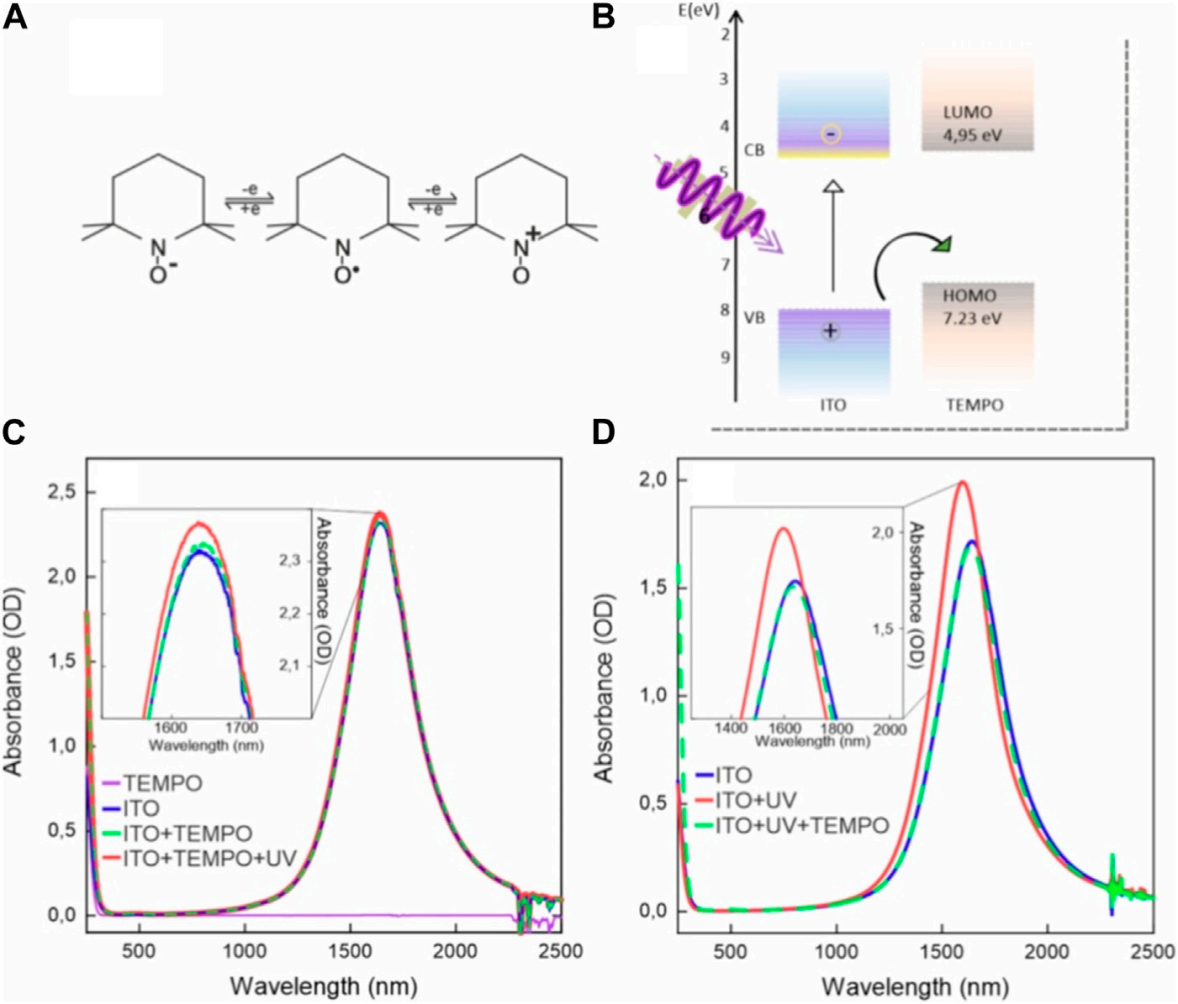
**(A)** Typical redox reactions of the TEMPO molecule (central molecule). **(B)** Energy alignment between ITO NC bands and TEMPO molecule energy levels. **(C)** A typical TEMPO absorption spectrum is shown in purple, adding no signatures in the region of the plasmon. The mixture of ITO and TEMPO (dashed green curve) displays the sum of the two spectra, indicating no ground state interaction between TEMPO and ITO NCs. After photodoping, a slight increase in the plasmon resonance intensity is observed (red curve). **(D)** To test the hypothesis that TEMPO can act as a hole and an electron acceptor, photodoping was performed in the absence of any hole scavenger. It resulted in the increase of LSPR peak intensity (from blue to red curves). Finally, the addition of 50 µL of the TEMPO stock solution resulted into the decrease in the plasmon peak back to its initial condition (green dashed curve).

TEMPO is a very well-known stable and versatile free radical molecule which can undergo reversible redox reactions (Figure 3A). Its main applications are its function as a catalyst in oxidation reactions (Wakui et al., 1999) and as a mediator in electrochemical reactions (Nutting et al., 2018; Ok et al., 2019). In the context of electrochemical systems, TEMPO serves as a mediator, facilitating charge transfer processes and promoting efficient electron transfer reactions (Nakatsuji et al., 1997; Sharma et al., 2023). Its redox potential, specifically the reversible TEMPO/TEMPO+ couple, allows it to act as a redox catalyst, participating in numerous electrocatalytic processes, including the oxidation of various organic substrates (Wakui et al., 1999; Ryan et al., 2019). TEMPO’s ability to undergo single-electron transfer reactions while remaining relatively inert to further reactions makes it a valuable tool for electrochemical investigations and applications, including in energy storage devices (Gatti et al., 2021), organic synthesis (Nutting et al., 2018), and sensors (Wu et al., 2020).

Considering the electrochemical reversibility and versatility of TEMPO, and its favorable energy-level alignment with the ITO NC band structure (Ok et al., 2019) (Figure 3B), TEMPO’s impact on the photodoping dynamics of ITO NCs was investigated. Two distinct experimental conditions were set in order to assess possible interactions of UV light on TEMPO and to assess whether it could act as an electron scavenger as well. In the first set of experiments, TEMPO was added to the ITO NC solution prior to illumination (Figure 3C). In the second set of experiments, TEMPO was added after illumination in absence of any hole scavenger (Figure 3D). Figure 3C displays the absorption spectra of a TEMPO solution in hexane (purple curve), ITO NC solution with TEMPO (green dashed curve), and photodoped ITO NCs with TEMPO (red curve). The LSPR peak of ITO NCs in this mixture does not exhibit significant changes in intensity or position before and after illumination. Indeed, after photodoping, the increase in peak intensity is only approximately 1.7%, which is significantly lower than the values for both EtOH-added or pristine ITO NC solutions, as reported in the previous section. For a better understanding of the role of TEMPO on the photodoping process, hence, we performed the reverse experiment, where TEMPO is added after illumination (Figure 3D). ITO NCs alone in solution demonstrate the ability to undergo photodoping, moving from the blue to the red curve in Figure 3D, as already reported in Figure 2B. Upon the addition of TEMPO, however (green dashed curve in Figure 3D), the position and intensity of the LSPR peak return to values comparable to those observed in the as-prepared state. The outcome of both experiments suggests that TEMPO is acting as an electron scavenger, rather than a hole scavenger. The addition of TEMPO before photodoping prevents the accumulation of electrons, as observed by a static LSPR position after light absorption. Furthermore, the addition of TEMPO after photodoping reverts the effect on LSPR, bringing the LSPR peak back to its initial position and intensity. These conclusions are supported by similar dynamics of LSPR peak position and intensity, as reported in the literature (Brozek et al., 2016; Ghini et al., 2021b; Shubert-Zuleta et al., 2023). Indeed, in these studies, the authors were investigating the effect of different electron scavengers on photodoped NCs. TEMPO electron scavenging ability, however, highlights the possible reversibility of the photodoping processes itself and a light-driven charging of TEMPO.

### Crystal violet

At this point, it is worth highlighting, again, how it is important that the addition of the hole scavenger to a solution containing ITO NCs does not compromise the chemical–physical stability of the system. In addition to the compatibility from from the solubility point of view solubility and, therefore, in terms of the solvent that has to be used to “host” both the nanoparticles and the redox species, it is necessary to verify that the presence of the new molecule does not activate processes of degradation or aggregation of the metal–oxide nanoparticles. This is the case, for example, of crystal violet, as illustrated in Supplementary Figure S3. This compound finds similar applications, as for the case of TEMPO and, also, as a ligand for other semiconductors (Jun et al., 2017), favoring charge transfer (Cañamares et al., 2008). In our case, however, the addition of crystal violet de-stabilizes the ITO NC solution, as evidenced by the decrease in the LSPR peak intensity and the subsequent broadening of the peak after photodoping.

### Ferrocene

**FIGURE 4:**
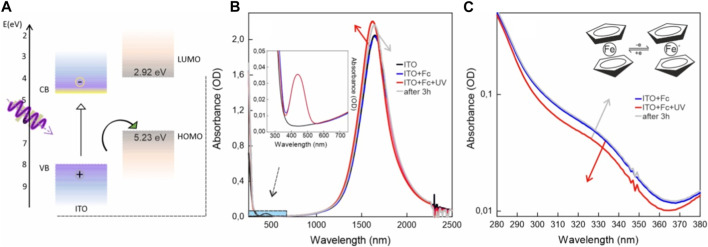
**(A)** ITO NC band structure compared to ferrocene energy-level alignment. **(B)** Absorption spectra of ITO NCs (black curve), ITO NCs mixed with ferrocene previously (blue dashed curve) and after photodoping (red curve), and the same system 3 h after illumination (gray curve). ITO NCs and ferrocene mixture consists of the sum of the two spectra, indicating no ground-state interactions. **(C)** Close-up of panel **(B)** grey in the UV range focusing on the changes in ferrocene absorption.

Finally, we wanted to investigate the possible exploitation of the ferrocene/ferrocenium system in combination with the ITO NC photodoping process. Ferrocene is a metal–organic compound consisting of two cyclopentadienyl anions (C5H5-) coordinated to a central iron (Fe^2+^) cation. It has diverse uses, such as in catalysis for various inorganic transformations (Lin et al., 2017). In electrochemical studies, ferrocene serves as a reference compound for potential measurements due to its well-defined, reversible redox couple (Fe^2+^/Fe^3+^), which enables accurate calibration and characterization of electrochemical systems (Pape et al., 2015). Moreover, the use of ferrocene in electrochemical energy storage devices, such as redox flow batteries and supercapacitors, showcases its potential as a promising candidate for advancing sustainable and high-performance energy technologies (Armstrong et al., 2020). Reversibility-based switching functions are also used in molecular electronic applications (Tahara et al., 2015; Karmakar et al., 2020). In addition, ferrocene and its derivatives have been used as ligands acting as molecular hole acceptors on photoexcited semiconductor quantum dots (Dorokhin et al., 2009; Ding et al., 2015; Dutta et al., 2019; Vogel et al., 2022). Their properties found application, for example, as reversible traps or redox mediators in photocatalysis (Olshansky et al., 2015; Olshansky et al., 2017; Li et al., 2021). Charge transfer processes were observed using the ferrocenium/ferrocene redox couple with perovskite NCs (Dubose and Kamat, 2019). Its use in electrochemistry and its favorable energy alignment (Manfredi et al., 2020) with the ITO band structure (Figure 4A) made ferrocene a good candidate for this study.

Figure 4B presents the photodoping results on ITO NCs and the ferrocene solution. First, as illustrated in Figure 4B, the spectra of ITO and mixed ITO-ferrocene show no remarkable difference in the position and intensity of the LSPR peak, indicating no interactions at the ground level. Upon illumination, under these conditions, ITO NCs undergo photodoping, as demonstrated by the blue shift and intensity increase of the LSPR peak (as indicated by the red arrow). Photodoping increases the intensity of the LSPR peak by 8.3%, improving its performances with respect to using only ITO without any hole scavenger. To assess the reversibility of the photodoping process, the cuvette was stored in an argon-filled glovebox for 3 h. Absorption spectra were then taken. Figure 4B shows how the photodoped ITO NC spectrum redshifts overtime and reduces in intensity after 3 h. For the sake of clarity, we included in Supplementary Figure S4 a zoomed-in view describing the changes in the LSPR peak. This ability of ITO NCs to recover pre-photodoping levels of LSPR peak intensity and position, even in an oxygen-free environment, might suggest the ability of oxidized ferrocene in extracting stored electrons in charged NCs. The typical absorption features, as illustrated in the inset of Figure 4B, confirm the presence of ferrocene in the systems. The ferrocene optical response is characterized by two main transitions, approximately at 322 nm and 442 nm (Paul et al., 2019), with a low molar extinction coefficient, whereas its oxidized form, ferrocenium, usually presents an absorption band shifted toward the red (approximately 600–700 nm). Figure 4C shows a close-up view of the UV range of the absorption spectra from Figure 4B. It displays the absorption spectra for ITO NCs mixed with the ferrocene solution previously, after photodoping and after 3 h (same color legend, as in Figure 4B). After photodoping, we observed a decrease in the contribution in the UV region of the absorption in correspondence of one of the peaks of ferrocene. Then, the spectral shape recovers after 3 h. This effect suggests a reversible mechanism of oxidation and reduction of ferrocene upon interaction with photodoped ITO NCs. We could not find any specific signature of the oxidized form of ferrocene in the red-NIR range. Assuming a similar or lower concentration of ferrocenium with respect to ferrocene, this effect could be ascribed to the lower extinction coefficient and the presence of the ITO NC-LSPR peak tail.

## Conclusion

We analyzed the possible use of different redox couples to further enhance the effect of photodoping in ITO NCs and develop a new hybrid system that is more convenient for energy applications and capable of undergoing cycles of re-use. For this purpose, we tested well-known compounds, TEMPO, crystal violet, and ferrocene. We studied the charge transfer interactions with ITO NCs depending on the photo-activation of the latter. As for the case of the TEMPO molecule, we observed how the presence of the redox mediator could induce a transfer of the photo-generated electrons from ITO, which can lead to the re-generation of the semiconductor. Compatibility is equally important from the energy perspective and the chemistry of the system to develop a stable hybrid solution, as evident in the experiment with crystal violet. Ferrocene has demonstrated to be an attractive alternative that can, in fact, support the increase in charge density of the doped metal oxide nanoparticles, offering hole-trapping channels. Moreover, the oxidation of ferrocene revealed a useful reversible characteristic, making use of the photo-generated charges. These results indicate an interesting new candidate for future applications like solar redox flow battery systems.

## Data Availability

The datasets presented in this study can be found in online repositories. The names of the repository/repositories and accession number(s) can be found at: https://doi.org/10.5281/zenodo.8304453.
