# Supplementary material for: Light-driven reversible charge transfers from ITO nanocrystals
Source: Front Chem. 2023 Nov 2;11:1288681. doi: 10.3389/fchem.2023.1288681 (PMC10652769; doi:10.3389/fchem.2023.1288681)
Supplement: Supplementary file 1 [file DataSheet1.docx]

Supplementary Material

Light-driven reversible charge transfers from ITO Nanocrystals

Luca Rebecchi^1,2^, Andrea Rubino^1^, Andrea Camellini^1,3^, Ilka Kriegel ^1*^

^1^ Functional Nanosystems, Istituto Italiano di Tecnologia, via Morego 30, 16163 Genova, Italy

^2^ Dipartimento di Chimica e Chimica Industriale, Università degli Studi di Genova, Via Dodecaneso 31, 16146 Genova, Italy

^3^ Department of Mechanical Engineering, Columbia University, New York, New York 10027, USA

*** Correspondence:**Ilka Kriegel
[ilka.kriegel@iit.it](mailto:ilka.kriegel@iit.it)


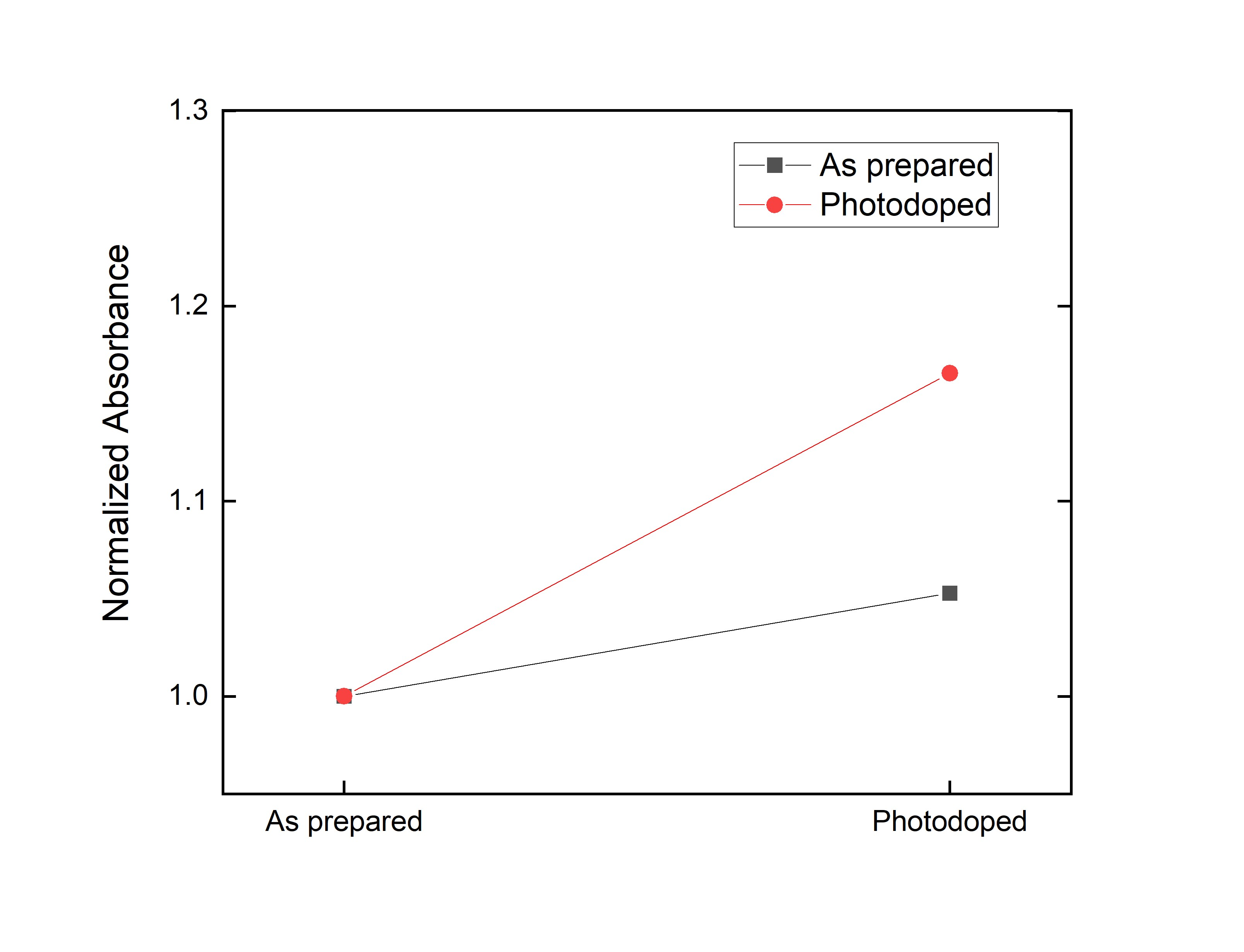


Figure S1 Comparison between the normalized LSPR absorbance in the as-prepared state and photodoped state, for ITO colloidal solution as is, or additioned with ethanol. In both case, we used 15 minutes of UV exposure time.


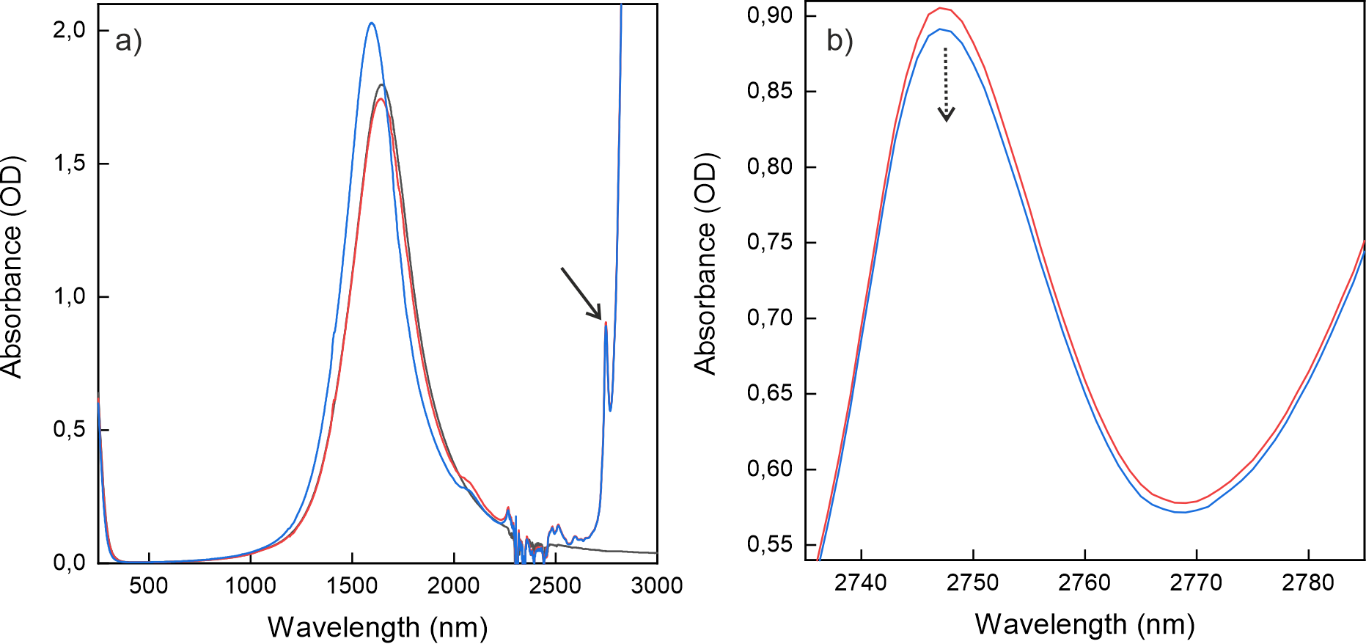


Figure S2 A) Absorption spectra of the ITO NC systems in containg EtOH: black curve corresponds to the solution with ITO NC; red curve corresponds to the same solution after EtOH addition; blue curve corresponds to the change after photodoping. B) zoom – in of the OH-stretching peak (red line) decreasing in intensity after photodoping (blue line).


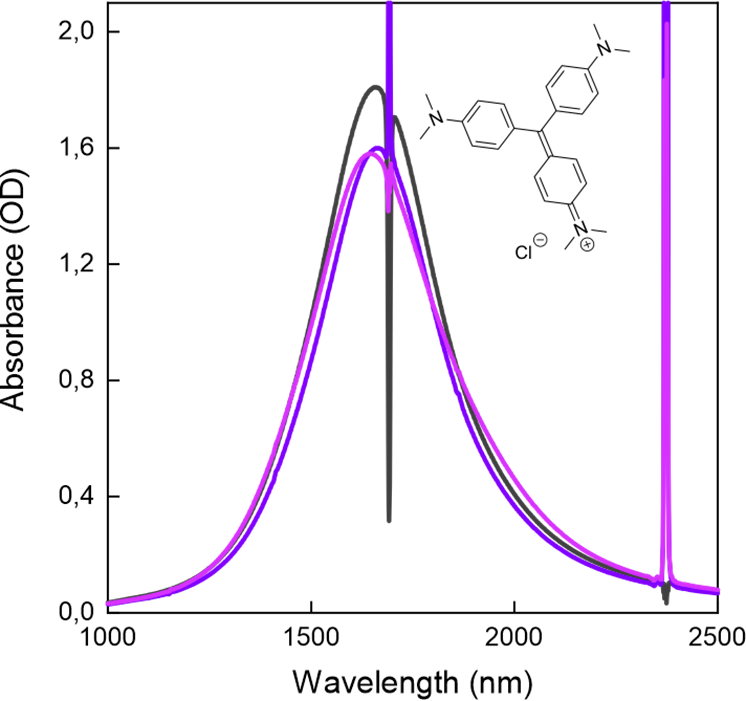


Figure S3 Absorption spectra recorder for the tests with crystal violet including ITO NC solution response (black curve), absorption change after crystal violet addition (purple curve) and after photodoping (pink curve).


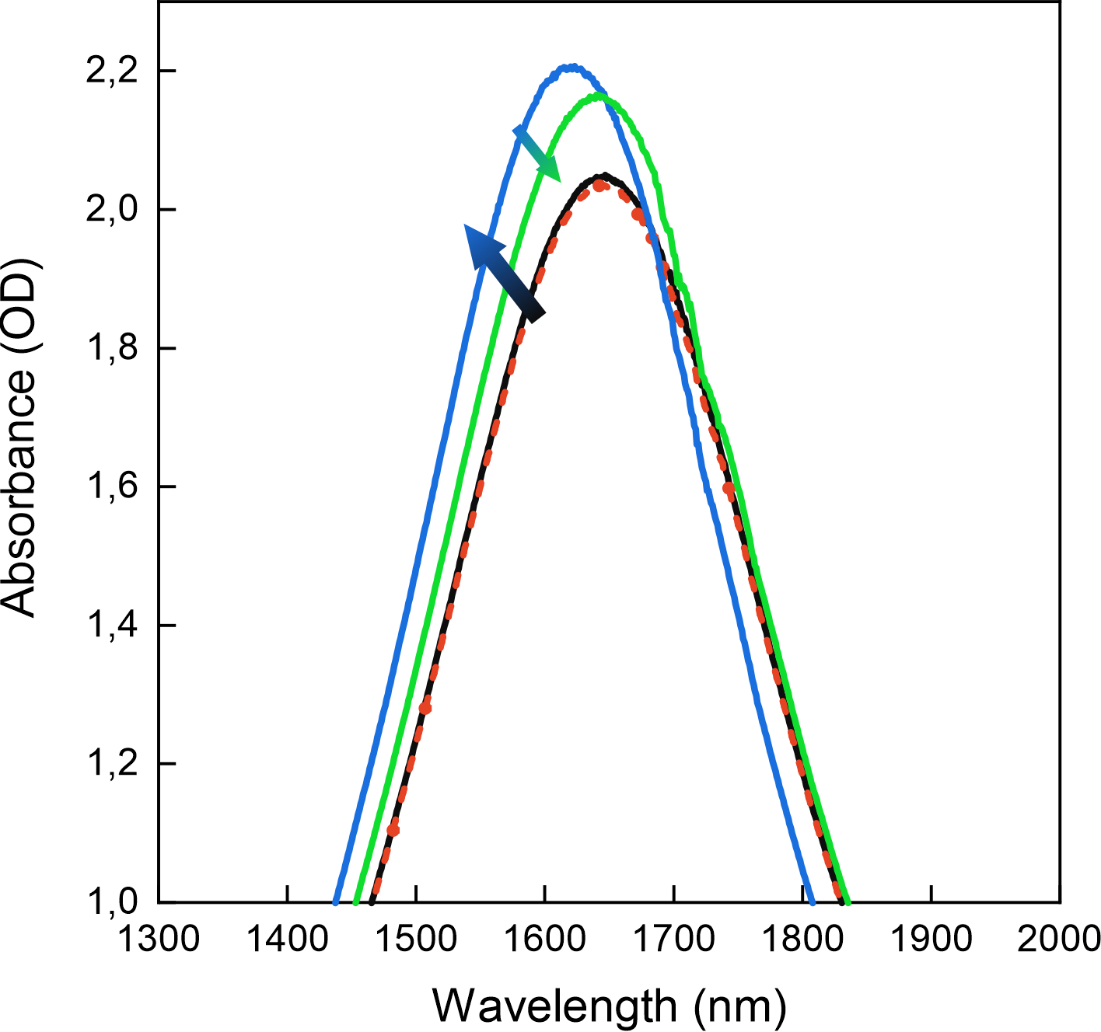


Figure S4 Zoom-in of the LSPR peaks from figure 4b. Absorption recorded for ITO NCs (black line) and ITO NCs mixed with ferrocene before (red dashed line) and after photodoping (blue line). The green curve corresponds to the LSPR peak of the photo-doped systems recorded 3h after illumination.
